# The development and improvement of immunodeficient mice and humanized immune system mouse models

**DOI:** 10.3389/fimmu.2022.1007579

**Published:** 2022-10-19

**Authors:** Jiaxuan Chen, Shuzhen Liao, Zengzhi Xiao, Quanren Pan, Xi Wang, Kangyuan Shen, Shuting Wang, Lawei Yang, Fengbiao Guo, Hua-feng Liu, Qingjun Pan

**Affiliations:** Guangdong Provincial Key Laboratory of Autophagy and Major Chronic Non-communicable Diseases, Affiliated Hospital of Guangdong Medical University, Zhanjiang, China

**Keywords:** immunodeficient mouse, humanized immune system mouse, nude mouse, NOD mouse, NOD/SCID mouse

## Abstract

Animal models play an indispensable role in the study of human diseases. However, animal models of different diseases do not fully mimic the complex internal environment of humans. Immunodeficient mice are deficient in certain genes and do not express these or show reduced expression in some of their cells, facilitating the establishment of humanized mice and simulation of the human environment *in vivo*. Here, we summarize the developments in immunodeficient mice, from the initial nude mice lacking T lymphocytes to NOD/SCID rg^null^ mice lacking T, B, and NK cell populations. We describe existing humanized immune system mouse models based on immunodeficient mice in which human cells or tissues have been transplanted to establish a human immune system, including humanized-peripheral blood mononuclear cells (Hu-PBMCs), humanized hematopoietic stem cells (Hu-HSCs), and humanized bone marrow, liver, thymus (Hu-BLT) mouse models. The different methods for their development involve varying levels of complexity and humanization. Humanized mice are widely used in the study of various diseases to provide a transitional stage for clinical research. However, several challenges persist, including improving the efficiency of reconstructing the human B cell immune response, extending lifespan, improving the survival rate of mice to extend the observation period, and improving the development of standardized commercialized models and as well as their use. Overall, there are many opportunities and challenges in the development of humanized immune system mouse models which can provide novel strategies for understanding the mechanisms and treatments of human disease.

## Immunodeficient mice

**Figure 1:**
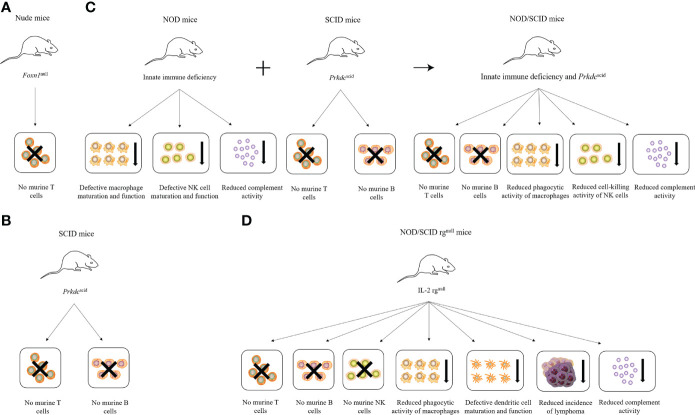
Characteristics of different immunodeficient mice. **(A)** Nude mice lack T cells due to *Foxn1* mutation. **(B)** SCID mice lack T and B cells due to *Prkdc* mutation. **(C)** NOD mice combined with SCID mice produce NOD/SCID mice, which lack T and B cells and have reduced phagocytic activity of macrophages, the cell-killing activity of NK cells, and complement activity. **(D)** NOD/SCID rg^null^ mice lack T, B, and NK cells and have reduced phagocytic activity of macrophages and complement activity, defective dendritic cell maturation and function, and reduced incidence of lymphoma due to the loss of the IL-2 receptor γ chain.

The development of immunodeficient mice occurred in four main stages. The first stage included nude mice that are simply deficient in T lymphocytes owing to abnormal thymus development (1). However, the application of nude mice in many diseases remains limited because of their low relative degree of immunodeficiency. The second stage included mice with severe combined immunodeficiency (SCID), carrying a mutation of the *Prkdc* gene (2, 3). SCID mice are deficient in T and B lymphocytes, but retain natural killer (NK) cells and show “leakage” (4). The SCID mutation was then introduced into non-obese diabetic (NOD) mice with NK cell defects to obtain NOD/SCID mice (5), forming the third stage of immunodeficient mice. However, these mice exhibit a high frequency of spontaneous thymic lymphoma and short life cycles, as well as partial NK cell activity. Therefore, their application as a humanized animal model has remained limited (5). To improve this situation, the fourth stage of immunodeficient mice, NOD/SCID rg^null^ mice, was developed by knocking out the IL-2 receptor gamma chain (IL-2 rg); these knock-out mice had a higher rate of human-cell implantation without leakage or spontaneous thymomas, and are currently the gold standard immunodeficient mouse model (6). The characteristics of different immunodeficient mice are summarized in **Figure 1**.

### Nude mice

Nude mice are the earliest immunodeficient mouse model, first reported by Flanagan in 1966 (1). Owing to an allele mutation on chromosome 11, a resultant defect in the *Foxn1* gene prevents normal thymus development (7), thereby leading to a mature T lymphocyte deficiency. The main immunoglobulin in these mice is IgM (8), with little or no IgA. As such, they do not show a rejection reaction to allogeneic tissue (9). The commonly used strains include BALB/c-nu, Swiss-nu, NC-nu, and NIH-nu, all of which are widely used in the study of immune diseases and tumors (10). However, as they still retain B cells and NK cells, they cannot completely accept human immune cell engraftment, and so cannot be used as an ideal humanized mouse model (11).

### SCID mice

In 1983, researchers found CB-17 inbred mice that carried a recessive mutation of a single gene on chromosome 16, which led to the abnormal recombination enzyme activity of the sequence encoding the mouse lymphocyte antigen receptor gene VDJ, due to which immunoglobulin, T, and B lymphocyte receptors could not be synthesized effectively (3). This mutation obstructs the repair and recombination of T and B cell receptors and seriously affects the differentiation and maturation of these cells, resulting in the lack of mature T and B cells and low immunoglobulin levels in the peripheral blood or lymphoid organs of SCID mice (2). However, the NK cells and macrophages in SCID mice function normally (12, 13). Furthermore, “leakage” was observed (4, 14, 15), meaning that 2 to 23 percent of the mice showed recovery of T and B lymphocytes with increasing age (16). As SCID mice are highly sensitive to radiation, Rag^null^ mice were generated by the knockout of recombinant activated genes Rag1 (15) or Rag2 (17) to reduce their radiosensitivity. Rag1 and Rag2 induce V(D)J rearrangement of TCR and immunoglobulin genes by producing DNA double-strand breaks. Homozygous mutations in these genes result in the inability to produce mature T and B cells and produce the same SCID-like phenotype (18). Similar to the SCID mutation, mice with the Rag mutations lack mature T and B lymphocytes. Contrastingly, this mutation does not repair spontaneously. Nevertheless, these mice also allow limited human cell and tissue engraftment because of highly active NK cells (19–21).

### NOD/SCID mice

In 1980, researchers obtained nonobese diabetic (NOD) mice *via* inbreeding and selective breeding, with pathological characteristics and changes similar to those in human diabetes (22). NOD mice have defects in their innate immune system, with low NK cells and macrophage activity, and an absence of circulating complement. Introducing the SCID mutation into the genetic background of NOD mice was hypothesized to result in NOD/SCID mice with simultaneously defective adaptive and innate immunity (23). Indeed, researchers successfully introduced the SCID mutation into NOD mice in 1995. The resulting NOD/SCID mice showed functional loss of T and B lymphocytes and other immune cells, as well as defective NK cell function, resulting in a higher degree of immune deficiency than in the previously noted mouse models (5). Human B cell reconstruction in nude mice and SCID mice was poor. In one study, NOD/SCID mice injected with 1×10^5^ human CD34^+^ cells showed that humanized B cells from different organs showed different stages of maturation, with immature IgM^-^IgD^-^ CD24^hi^ CD38^hi^ B cells predominating in the bone marrow and mature CD5^+^ IgM^+^ IgD^+^ CD24^int^ CD38^int^ CD19^+^ B cells predominating in the spleen and peripheral blood.

Compared with SCID mice, human tumors and immune cells had better survival status in NOD/SCID mice (23). The NOD/SCID mice had the following characteristics (1): low NK cell levels, with significantly reduced killing function; (2) complement C5 deficiency, resulting in inhibition of complement activation;(3) defective IL-1 secretion in lipopolysaccharide-induced macrophages. These characteristics enabled the generation and survival of human cells and grafts in NOD/SCID mice at higher levels. However, this model remained unsuitable, owing to certain defects, including radiosensitivity, which only allows a small radiation dose. T and B cell leakage also occurred in older mice, and their average life span was only 8 months. Furthermore, the NOD gene mutation in NOD/SCID mice increased the probability of spontaneous thymic lymphoma, resulting in a short life cycle of such mice along with partial NK cell activity, limiting its application as a humanized animal model (5).

NOD/SCID mice are not as commonly used to generate humanized mice because they require a higher dose of HSCs for efficient engraftment, compared with more deficient mouse strains like NOD/SCID rg^null^ mice and they developed thymic lymphomas shortening their lifespan. Despite these disadvantages, the model is still in use because of its unique characteristics. For example, it has been shown that NOD/SCID mice better support the development of human gut-associated lymphoid tissue (GALT) structures due to the presence of the common gamma chain. Therefore, when more robust human GALT structures are needed, NOD/SCID BLT mice may be preferred (24). One study also showed enhanced human cell reconstitution in the GALT of BLT mice. This study, using HIV infection of humanized mice (BLT) as a model of heterosexual transmission, demonstrated that blocking lymphocyte egress from lymph nodes prevented viremia and infection of the gut (25).In addition, NOD/SCID mice transplanted with HSCs are specifically used to generate mice that possess human myeloid and B cells but are devoid of human T cells following the transplant to study certain aspects of EBV and HIV infection (26, 27).

### NOD/SCID rg^null^ mice

The IL-2 receptor gamma chain, also known as the common cytokine receptor gamma chain, is a key component of high-affinity receptors for cytokines such as IL-2, IL-4, IL-7, IL-9, IL-15, and IL-21. The development and maturation of T and B lymphocytes and NK cells require the participation of some of these cytokines. The loss of the IL-2 receptor gamma chain hinders the development of T and B lymphocytes as well as NK cells and severely weakens the innate and adaptive immune systems of mice (28). When IL-2 rg^null^ was combined with SCID, Rag1^null^, or Rag2^null^ mutations, the resulting NOD/Shi-SCID IL-2^null^ (NOG) (29), NOD/LtSz-SCID IL-2^null^ (NSG) (30), and NOD-Rag1^null^ IL-2 rg^null^ (NRG) (31) mice were deficient in T and B lymphocytes as well as NK cells (13). These mice completely lost the ability to mount an adaptive immune response and showed serious defects in the innate immune system; which are the main requirements for immunodeficient mice for the construction of humanized mouse models (6).

NOD/SCID rg^null^ mice can be divided into NOG and NSG mice according to the mutation of the IL-2 receptor gamma chain. The major difference between the NSG and NOG strains is that the IL-2 rg targeted mutation used to develop the NSG strain is a complete null so that no IL-2 rg is expressed, effectively hindering cytokine binding, whereas the IL-2 rg mutation in the NOG strain produces a protein that is expressed and will bind cytokines but cannot transduce the signal (32). NOG and NSG mice were found to be the best models for human cell and tissue transplantation, with a higher transplantation success rate than either SCID or NOD/SCID mice (33, 34). Another important advantage of NOG and NSG mice is the absence of leakage and spontaneous thymomas, which may also be related to the lack of active IL-2 rg. Moreover, several immunodeficient mouse strains, such as NSGB2m and NSG-SGM3, have been derived by gene modification based on NSG mice. These mice are more advantageous in xenotransplantation (35, 36). One study comparing the implantation rate of human cells in the peripheral tissues of NSG mice with that in NOD/SCID mice showed a significantly higher implantation rate of human cells in NSG and NOG mice than in NOD/SCID mice. In addition, the implantation rate of human cells in the bone marrow of NSG mice was higher than that in the other strains, especially in females (37). Therefore, NSG mice are good candidates for generating humanized immune system mouse models. Another study examined the recovery of the immune system in humanized mice after the transplantation of human hematopoietic stem cells in NSG mice. The results showed that T, B cells, monocytes, macrophages, and neutrophils were developed to normal human levels in these mice. Moreover, the phagocytic ability of monocytes and macrophages, and the secretion ability of inflammatory factors under TLR4 stimulation also developed to normal human levels (38).

Signal regulatory protein α (SIRPα) is a transmembrane protein that contains three Ig-like domains within the extracellular region. SIRPα is expressed in macrophages, myeloid cells, and neurons, and interacts with its ligand CD47 *via* respective IgV-like domains, where the NOD strain has specific polymorphism. CD47 is a member of the immunoglobulin (Ig) superfamily that is ubiquitously expressed in hematopoietic as well as non-hematopoietic cells. The cytoplasmic region of SIRPα has immunoreceptor tyrosine-based inhibitory motifs (ITIMs), and the cell surface binding of CD47 with SIRPα on macrophages provokes inhibitory signals *via* phosphorylation of ITIM of SIRPα (39), preventing their phagocytic activity (40–42). A recent study showed that transgenic expression of mouse CD47 into the CD34^+^CD38^-^ human fetal liver cells significantly enhanced the human cell engraftment into BALB-RG mice (43). Based on these results, it is assumed that the binding of NOD-SIRPα with human CD47 produces signals for mouse macrophages not to engulf human HSCs, presumably making the strain permissive for human HSC engraftment (44). The important question was whether the NOD-specific highly efficient human cell engraftment *in vivo* could be explained solely by the NOD-SIRPα polymorphism. In one study, Yamauchi et al. established a C57BL/6-Rag2^null^IL-2rg^null^ (C57BL/6-RG) line harboring the NOD-type SIRPα. The results clearly show that the replacement of the C57BL/6-type SIRPα with the NOD-type SIRPα is sufficient for the C57BL/6-RG strain to be endowed with the xenotransplantation capability at least equal to NOD-RG mice. Thus, they successfully segregated the genetic abnormality responsible for efficient human cell engraftment from multiple genetic abnormalities in the NOD strain (45). The simplified humanized mouse system established by the new C57BL/6-Rag2^null^IL-2rg^null^NOD-SIRPα (BRGS) strain should be very useful to improve xenotransplantation strategies in studies on human cell biology. In one study, Di Santo et al. induced the expression of thymic-stromal-cell-derived lymphopoietin (TSLP) in a BALB/c Rag2^-/-^IL-2rg^-/-^SIRPα^NOD^ (BRGS) human immune system (HIS) mouse model. The resulting BRGST HIS mice developed a full array of LNs with compartmentalized human B and T cells. Compared with BRGS HIS mice, BRGST HIS mice have a larger thymus, more mature B cells, and abundant IL-21-producing follicular helper T (TFH) cells, and show enhanced antigen-specific responses. Peripheral human B cells in HIS mice retain an immature, transitional phenotype with elevated expression of CD24 and CD38. In BRGS and BRGST HIS mice, they observed this predominant population of CD24^hi^CD38^hi^ immature B cells in the bone marrow, liver, and spleen. In contrast, mature CD24^lo^CD38^lo^ cells were the dominant human B cell subset in LNs of BRGST mice. Although they did not observe notable differences in these different B cell subsets between the two models, the total numbers of mature CD24^lo^CD38^lo^ B cells in LNs were significantly increased in BRGST HIS mice compared with those in BRGS mice (46).

Humanized mouse models constructed by engrafting peripheral blood mononuclear cells have mainly revealed the presence of human T cells (47, 48). However, in stem cell transplant models, B-cell reconstitution is efficient with T-cell engraftment lagging (49). Although both HSC-infused newborn and adult mice were highly reconstituted with human B cells, the development of B cells was arrested in an early stage and did not suffice for reconstitution of human immunoglobulins (natural antibodies) in serum, other than IgM (50). Impairment of human T and B cell function in HSC reconstituted IL-2 rg^null^ genetic stocks has been attributed to the lack of expression of human leukocyte antigens (HLA) in the mouse thymus since HLA molecules are required for the development of human T cells that in turn, are essential for stimulation of B cells towards immunoglobulin class switching and antibody secretion (51, 52). In one study, Danner et al. generated NOD.Rag1KO.IL2RγcKO mice expressing HLA-DR4 molecules under the I-E^d^ promoter infused as adults with HLA-DR-matched human hematopoietic stem cells generating a new strain of NOD.Rag1KO.IL2RγcKO mice expressing HLA-DR*0401 molecules (DRAG mice). The presence of these HLA-DR4-IE transgenes allows irradiated DRAG mice to be engrafted with HLA-DR-matched hematopoietic stem cells; resulting in humanized T-cell and B-cell populations. The HLA-DR4 expressing mice reconstituted serum levels (natural antibodies) of human IgM, IgG (all four subclasses), IgA, and IgE comparable to humans, and elicited high titers of specific human IgG antibodies upon tetanus toxoid vaccination (53). In another study, Ito et al. generated transgenic mice with HLA-DRA-IEα and HLA-DRB1*0401-IEβ chimeric genes. The HLA-DRA-IEα/HLA-DRB1*0401-IEβ molecules rescued the development of CD4^+^ T cells in major histocompatibility complex (MHC) class II-deficient mice, but T cells expressing Vβ5, Vβ11, and Vβ12 were specifically deleted (54).

These various types of mice are suitable for constructing various humanized mouse models for studying tumors, hematological diseases, infectious diseases, immune diseases, and metabolic diseases (29).

## Development of humanized immune system mice

**Figure 2:**
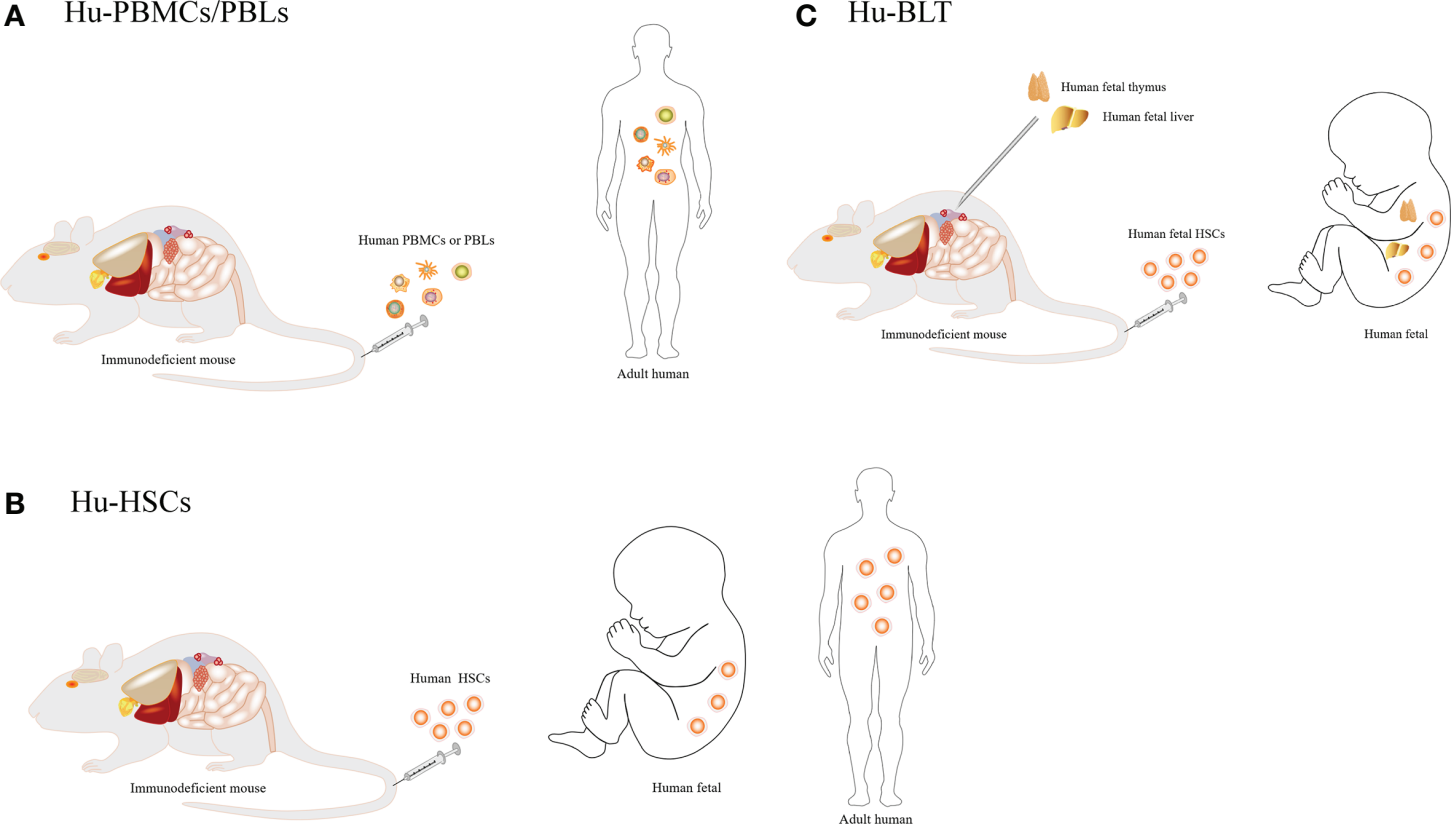
Different construction methods of humanized mice. **(A)** Injection of human peripheral blood mononuclear cells or lymphocytes. **(B)** Injection of human CD34^+^ hematopoietic stem cells. **(C)** Co-transplantation of human fetal thymus and fetal liver into the renal capsule of mice, involving injection of hematopoietic stem cells from the fetal liver or bone marrow of the same individual into mice.

Humanized immune system mice can be divided into three groups according to the method used for immune system reconstruction: humanized-peripheral blood mononuclear cells (Hu-PBMCs) or humanized-peripheral blood lymphocytes (Hu-PBLs), humanized hematopoietic stem cells (Hu-HSCs) and humanized bone marrow, liver, thymus (Hu-BLT) mouse models. The different construction methods and characteristics of humanized mice are shown in **Figure 2** and **Table 1**.

**Table 1:** Different construction methods and characteristics of humanized mice.

Models	Construction methods	Advantages	Disadvantages
Hu-PBMCs/PBLs	Injection of human peripheral blood mononuclear cells or lymphocytes	1 Sample is easy to obtain and the transplantation method is simple 2 Efficient and stable transplantation of T cells	1 Lack of B, NK, and other immune cells 2 Possible induction of GVHD 3 The massive injection of human cells results in EBV-associated lymphoproliferative disease
Hu-HSCs	Injection of human CD34^+^ hematopoietic stem cells	1 Multiple line of hematopoietic cell development, including T, B, myeloid, and NK cells 2 Less GVHD	1 Limited sample sources 2 Lack of T cells (NOD/SCID mice)
Hu-BLT	Co-transplantation of human fetal thymus and fetal liver into the renal capsule of mice, involving injection of hematopoietic stem cells from the fetal liver or bone marrow of the same individual into mice	1 Better T, B, myeloid, and NK cell development 2 Can produce the human mucosal immune system and secondary lymphoid tissue and mount an adaptive immune response 3 Human T cells are educated on human MHC (HLA-restricted) in the human thymic organoid	1 Limited sample sources 2 Possible induction of GVHD

Hu-PBMCs/PBLs, humanized-peripheral blood mononuclear cells/peripheral blood lymphocytes; Hu-HSCs, humanized hematopoietic stem cells; Hu-BLT, humanized bone marrow, liver, thymus; GVHD, graft-versus-host disease.

### Hu-PBMCs/PBLs mouse model

In this model, human peripheral blood mononuclear cells (PBMCs) or peripheral blood lymphocytes (PBLs) are transplanted into mice *via* a caudal vein or peritoneal injection (48, 55, 56). In general, 50–80% of human CD45^+^ cells can be detected in the blood and spleen of mice. Human CD3^+^ T cells are usually detected in the first week after transplantation, forming an ideal model for studying mature effector T cells (57). Small numbers of B cells, myeloid cells, and other immune cells are also detected in these mice (57, 58). In this model, human memory B cells can produce antibodies after antigen stimulation, but cannot produce a primary immune response. Human immune cells can survive for several weeks after transplantation and are effective to some extent. They can be efficiently infected with HIV, HBV, EBV, HSV, HCMV, KSHV, etc., and play an important role in allogeneic immune response and viral immunity studies (59–65). In the early stage of establishing the humanized mouse model, human peripheral blood mononuclear cells or peripheral blood lymphocytes were injected into nude mice. Since nude mice are only deficient in T lymphocytes, they cannot completely accept human PBMCs or PBLs, resulting in immune rejection (2). In later work, PBLs were directly transplanted into SCID mice to construct a humanized PBLs-SCID mouse model. In this model, a multi-lineage humanized immune system can be obtained, and long-term reconstruction can be maintained. However, the main problem with this model is the fatal graft-versus-host disease (GVHD) caused by the MHC mismatch between human T cells and mouse immune cells (35, 66). GVHD symptoms usually appear 4–6 weeks after injection of human PBMCs, with a short observation window of limited use (58, 67). However, this window can be prolonged and ameliorated by using NSG (NOD/SCID IL-2Rγ C^-/-^) or RG (BALB/C Rag2^-/-^ IL-2R γ C^-/-^) mice with deletion of the MHC-I or II genes (68). The Hu-PBLs model is the simplest and most economical humanized mouse model because of the easy availability of human PBLs. However, it also has several shortcomings, such as the low and unstable level of human lymphocyte reconstruction, lack of a normal lymphoid tissue structure and a follicular germinal structure in the spleen, EBV-associated lymphoproliferative disease after massive injection of human cells, and xenograft rejection.

### Hu-HSCs mouse model

In this model, the immunodeficient mice were irradiated with a sublethal dose to destroy the hematopoietic function of the autologous bone marrow in mice; human CD34^+^ hematopoietic stem cells (HSCs) were then transplanted into these immunodeficient mice through the vein or femoral artery (HSCs *via* intrahepatic injection as pups and *via* tail vein injection as adults) (69–71). Human HSCs in mice (such as NOG, NSG, etc.) developed into T cells, B cells, and NK cells, and formed bone marrow sources of inhibitory myeloid-derived suppressor cells (MDSCs), and other immune cells (72). The number of human CD45^+^ T cells usually reaches 25–60% in peripheral blood at 4 weeks after implantation (72). As these immune cells develop from transplanted HSCs and are tolerant to the mouse host, GVHD usually does not occur. The stable period can be as long as 10–12 weeks (73), allowing the study of HIV (74), EBV (75), other infection models, and hematopoietic system development. HSCs can be obtained from bone marrow, umbilical cord blood, or peripheral blood after mobilization using granulocyte colony-stimulating factor (G-CSF) (76). In the early stage of developing this model, human CD34^+^ hematopoietic stem cells were transplanted into NOD/SCID mice, which could reconstruct lymphocyte proliferation, but the resulting T cells were dysplastic. NSG, RG, or NOG (NOD/Shi-SCID IL-2Rγ C^-/-^) mice now allow better implantation of human cells (77, 78).

### Hu-BLT mouse model

The precursor of this model was the SCID-hu mouse model, which involved surgical implantation with a human fetal thymus and liver under the renal capsule of SCID mice (79, 80). However, this model had obvious shortcomings, such as a low level of human cell reconstruction, unstable development of human T cells, and short survival time (81). Human fetal thymus was also transplanted into SCID-hu mice; and although human T cells developed in the thymus, the level of humanized development was low and unstable. However, when human HSCs were transferred into the hu-HSCs mouse model, a large number of B cells and myeloid cells were generated, but human T cells were completely lacking. In one study, the advantages of SCID-hu mice and Hu-HSCs mice were combined. Human fetal thymus and fetal liver tissue blocks were transplanted from NOD/SCID mice irradiated with a sublethal dose under the renal capsule, and CD34^+^ HSCs were isolated from the homologous fetal liver by tail vein injection to construct Hu-BLT mice (82). The major difference between the BLT and the SCID-hu mouse model is the additional reconstruction of hematopoietic stem cells from the same fetal liver in the BLT model. The complete range of T cells, B cells, NK cells, DCs, monocytes, macrophages, and other human immune cells can be found in Hu-BLT mice *in vivo*. Furthermore, they can produce a human adaptive immune response, thereby constituting the most effective mouse model of human immune system reconstruction (83).

The Hu-BLT mouse model (NOD/SCID mice) also shows a human mucosal immune system, and secondary lymphoid tissue, and mounts adaptive human immune responses, such as the production of IgM, IgG, and other immunoglobulins. Therefore, the immune response of humanized BLT mice to implanted exogenous tissues or cells is more similar to the natural response of the human body. NOD/SCID mice were used in the early BLT model, whereas NSG, NOG, or RG mice are used in the improved model (82, 84). The use of human grafts can result in the production of more T cells, B cells, macrophages, NK cells, and DCs. In one study, the generation of humanized BLT mice by the co-transplantation of human fetal thymus and liver tissues and CD34^+^ fetal liver cells into NOD/SCID rg^null^ mice allows for the long-term reconstitution of a functional human immune system, with human T cells, B cells, dendritic cells, and monocytes/macrophages repopulating mouse tissues (85). As T cells in human thymus tissue have a high affinity for the MHC of mice, the Hu-BLT model may exhibit a graft-versus-host reaction after 20 weeks of implantation. Humanized mice constructed in the TKO-BLT model, (Rag2, IL-2YC, and CD47 triple gene knockout) did not develop GVHD at 45 weeks, and showed a better effect than the existing BLT mouse model (86).

BLT humanized mice are now widely used for studying tumors, immunology, infectious diseases, regenerative medicine, stem cell therapy, and other research areas (87–89). BLT mice have made a lot of contributions to the study of HIV infection. Using HIV vaginal infection of humanized mice as a model of heterosexual transmission, Deruaz et al. demonstrate that blocking the ability of leukocytes to respond to chemoattractants prevented HIV from leaving the female genital tract (25). In one interesting study, intravital microscopy was used to observe changes in humanized mice after the intervention. Usmani et al. show by intravital microscopy in humanized mice that perturbation of the actin cytoskeleton *via* the lentiviral protein Nef, and not changes to chemokine receptor expression or function, is the dominant cause of dysregulated infected T cell motility in lymphoid tissue by preventing stable cellular polarization required for fast migration (90). Smith et al. have developed a method to quickly propagate established BLT mice by the secondary transfer of bone marrow cells and human thymus implants from BLT mice into NSG recipient mice. In this way, they were able to expand one primary BLT mouse into a colony of 4–5 propagated BLT mice in 6–8 weeks. These propagated BLT mice reconstituted human immune cells, including T cells, at levels comparable to those of their primary BLT donor mouse. They also faithfully inherited the human immune cell genetic traits from their donor BLT mouse, such as the HLA-A2 haplotype that is of special interest for studying HLA-A2-restricted human T cell immunotherapies. This method provides an opportunity to overcome a critical hurdle to utilizing the BLT humanized mouse model and enables its more widespread use as a valuable preclinical research tool (91). Vatakis et al. used the BLT humanized mouse as a stem cell-based gene therapy tumor model. They use genetically modified human HSCs to construct the thymus/liver implant followed by injection of transduced autologous human HSCs. This approach results in the generation of genetically modified lineages. After the intervention, the regression of the tumor was observed by positron emission tomography (PET) (92). In conclusion, the BLT mouse model has many advantages in human disease research, but its complex construction process needs to be further optimized.

## The improvement of humanized immune system mouse models

The humanized mouse models can be further improved by irradiation or chemical pre-treatment, deletion of mouse autoimmune cells, injection of human cytokines, construction of viral vectors, high-pressure injection of gene expression plasmids, and construction of genetically engineered mice. The methods for improving humanized immune system mouse models are summarized in **Table 2**.

**Table 2:** Improvement of humanized immune system mouse models.

Treatment	Results	Reference
Irradiation and chemical reagents	Busilvex (35 mg/kg) → Mouse immune system ↓ Irradiation (2–3 Gy) → Mouse immune system ↓	(66) (93)
Knock out of mouse autoimmune cells	Anti-asialo GM1 antibody → Mouse NK cells ↓ CD122 antibody → Mouse NK cells ↓ IL-2R antibody → Mouse NK cells ↓ Cl2MDP → Mouse macrophages ↓	(29) (94) (94) (94)
Injection of human cytokines	Human G-CSF → Human dendritic cells, monocytes, and neutrophils ↑ Human IL-7 → Multi-lineage human cell differentiation Human SCF, IL-3, GMCSF, TPO → Human myeloid cells and lymphocytes ↑ Human FLT3L → Human dendritic cells ↑	(38) (95) (96) (97)
Construction of viral vectors	Adenoviral vectors overexpress human IL-15 → Human NK cells ↑ Lentiviral vectors overexpress human IL-7 → Human T cells and B cells ↑	(98) (99)
Injection of gene expression plasmids	Human IL-15 and Flt3l gene expression plasmid → Human NK cells ↑	(100)
Genetic engineering	Human M-CSF gene knock-in → Human monocytes and macrophages ↑ Human M-CSF, IL-3, GM-CSF, TPO gene knock-in →Humanized myeloid and NK cells ↑ Human SIRPαand TPO gene knock-in → Human hematopoietic engraftment levels ↑ Human TPO gene knock-in → Multi-lineage human immune cells and platelets ↑ Human SIRPα gene knock-in → Phagocytosis of macrophages ↓ Human SCF, GM-CSF, and IL-3 gene knock-in → Human myeloid cells ↑ Human SF, IL-3, and GM-CSF gene knock-in → Humanized myeloid cells ↑ Human SCF and KITL gene knock-in → Humanized myeloid cells ↑ Human M-CSF, IL-3, GM-CSF, TPO, SIRPα gene knock-in → Human myeloid and NK cells ↑ Human M-CSF, IL-3, SIRPα, TPO, GM-CSF, and IL-6 gene knock-in →More susceptible to SARS-CoV-2 infection	(41) (101) (102) (103) (104) (105) (106) (106) (107) (108)

“↑/↓” in humanized immune system mouse models represent an increase or decrease respectively compared with non-intervention control.

### Irradiation or chemical pretreatment

Immunodeficient mice could be irradiated or pretreated with chemical reagents to provide more “space” for humanized construction. One study compared the efficiency of transplantation and found that human immune cells could survive better in NOD/SCID mice when 2–3 Gy pre-radiation was performed before injection of human HSCs (93). Furthermore, a single dose (35 mg/kg) of Busilvex can achieve the same transplantation effect as 3.5 Gy irradiation (66). Therefore, these pretreatments may result in increased concentrations of growth factors and chemoattractants and reserve a certain amount of space for the development of human HSCs and immune cells in immunodeficient mice.

### Depletion of auto-immune cells in mice

The innate immunity of immunodeficient mice limits the regeneration of human immune cells. Mouse NK cells can be depleted by using CD122 or IL-2R antibodies (94). Another approach to deplete NK activity is the use of anti-asialo GM1 antibody injection before HSC transfer (29). Liposome-encapsulated dichloroethylene diphosphonate (Cl_2_MDP) can deplete mouse macrophages and facilitate better reconstruction of the human immune system (94, 109). Therefore, when a particular cell type needs to be focused, specific elimination of antibodies may be a good choice.

### Injection of human cytokines

With the development of immunodeficient mice and the improvement of reconstitution levels, the role of cytokines has attracted wide attention. In one study, a significant increase in neutrophils, monocytes, and DCs was obtained by injecting human G-CSF into NOG mice (38). Injection of human IL-7 into NOG mouse models was found to promote multi-lineage cell differentiation, achieving a reconstruction effect equivalent to that obtained with umbilical cord blood stem cells (95). In NOD/SCID mice injected with human SCF, IL-3, GMCSF, and TPO for two weeks, the development and differentiation of lymphocytes and myeloid cells were significantly improved (96). Furthermore, in NOD/SCID mice injected with human FLT3L, the number and function of DCs were significantly increased after four weeks (97). In summary, cytokines can greatly promote the construction of humanized mice. Further studies should focus on finding more suitable cytokines for the construction of humanized mice.

### Construction of viral vectors

With advances in molecular biology, viral vectors have become a common tool, which can transfer the required genetic material into cells, to achieve the effect of foreign gene expression. In one study, injection of human IL-15 or overexpression of human IL-15 using adenoviral vectors was found to promote NK cell development and maturation (98). Lentiviral vectors carrying the human IL-7 gene have been used to overexpress human IL-7 in Rag2^-/-^ γ C^-/-^ mice; the serum level of human IL-7 was maintained at a high level during the observation period of up to six months in these mice. Il-7 overexpression significantly increased the proportion of T and B cells in peripheral blood, but had little effect on the overall immune reconstitution and did not affect the differentiation of T cell subsets (99).

### Injection of gene expression plasmids

The high-pressure injection is a common technique for gene overexpression *in vivo*. A study on humanized mice generated *via* high-pressure injection of IL-15 and Flt3l expression vector found that the reconstruction of NK cells was significantly increased. Furthermore, these NK cells showed normal expression of activated receptors and inhibitory receptors, which could be induced to cause liver damage and could kill target cells *in vitro*, demonstrating that the reconstructed NK cells were functional (100).

### Genetic engineering

Mouse models genetically engineered from immunodeficient mice are more stable. Mice repopulated with human hematopoietic cells are a powerful tool for the study of human T and B cells *in vivo*. However, existing humanized mouse models are unable to support the development of human innate immune cells, including myeloid cells and NK cells. In one study, Rongvaux et al. describe a mouse strain, called MI(S)TRG, in which human versions of four genes (human M-CSF, IL-3, GM-CSF, and TPO) encoding cytokines important for innate immune cell development are knocked into their respective mouse loci. The human cytokines support the development and function of monocytes/macrophages and natural killer cells derived from human fetal liver or adult CD34^+^ progenitor cells injected into the mice. Human macrophages infiltrated a human tumor xenograft in MI(S)TRG mice in a manner resembling that observed in tumors obtained from human patients (101).

Human CD34^+^ hematopoietic stem and progenitor cells (HSPCs) can reconstitute a human hemato-lymphoid system when transplanted into immunodeficient mice. Although fetal liver-derived and cord blood-derived CD34^+^ cells lead to high engraftment levels, engraftment of mobilized, adult donor-derived CD34^+^ cells has remained poor. Saito et al. generated so-called MSTRG and MISTRG humanized mice on a Rag2^-/-^IL-2rg^-/-^ background carrying a transgene for human SIRPα and human homologs of the cytokine macrophage colony-stimulating factor, TPO, with or without IL-3 and granulocyte-macrophage colony-stimulating factor under murine promoters. They transplanted mobilized peripheral blood (PB) CD34^+^ cells in sublethally irradiated newborn and adult recipients. Human hematopoietic engraftment levels were significantly higher in bone marrow (BM), spleen, and PB in newborn transplanted MSTRG/MISTRG recipients as compared with non-obese diabetic/severe combined immunodeficient IL-2rg^-/-^ or human SIRPα-transgenic Rag2^-/-^ IL-2rg^-/-^ recipients. Furthermore, newborn transplanted MSTRG/MISTRG mice supported higher engraftment levels of human phenotypically defined HSPCs in BM, T cells in the thymus, and myeloid cells in non-hematopoietic organs such as liver, lung, colon, and skin, approximating the levels in the human system. Similar results were obtained in adult recipient mice (102).

In addition, in one study, human TPO knock-in mice were constructed using Rag2^-/-^γ C ^-/-^ mice, resulting in an increased level of humanized reconstruction, multi-lineage immune cell development and differentiation, and increased platelet counts (103). SIRPα inhibits the phagocytosis of macrophages physiologically (110) and plays an important role in the maintenance of hematopoietic stem cells, red blood cells, and platelets (101). In one study, the phagocytic activity of macrophages was significantly inhibited by knock-in human SIRPα in Rag2^-/-^ γ C^-/-^ mice (104). In another study, the expression of human monocytes and macrophages in bone marrow, spleen, peripheral blood, lung, liver, and the abdominal cavity was significantly increased by knock-in of human M-CSF into Rag2^-/-^ γ C^-/-^ mice and their migration, phagocytosis, and activation were enhanced (41). Human SCF, GM-CSF, and IL-3 have also been expressed in NSG mice using transgenic technology, to form NSG-SGM3 mice. The reconstruction level of myeloid cells, especially dendritic cells, is significantly improved in these mice (105). In addition, NSG-3GS mice were also constructed by knock-in of human SF, IL-3, and GM-CSF into NSG mice. Humanized myeloid cells were significantly increased in these mice (106). Similarly, myeloid cells were significantly increased by the knock-in of human SCF and KITL in NSG mice (106). On this basis, a study was conducted integrating human M-CSF, IL-3/GM-CSF, TPO, and SIRPα in Rag2^-/-^ γ C^-/-^ mice which promoted the increase of human myeloid and NK cells (107).

Humanized mice are also irreplaceable in COVID-19 research. Severe COVID-19 is characterized by persistent lung inflammation, inflammatory cytokine production (111–113), viral RNA, and sustained interferon (IFN) response all of which are recapitulated and required for pathology in the SARS-CoV-2 infected MISTRG6-hACE2 humanized mouse model (based on the Rag2^-/-^ IL2rg^-/-^129xBalb/c background supplemented with genes for human M-CSF, IL-3, SIRPα, TPO, GM-CSF, and IL-6 knocked into their respective mouse loci) of COVID-19 with a human immune system (108). In this study, Sefik et al. show that SARS-CoV-2 infection and replication in lung-resident human macrophages is a critical driver of the disease (108). In summary, the genetic engineering of humanized mice plays a unique role in modeling and studying specific diseases.

## Conclusions and future prospects

To the present day, immunodeficient mice have undergone development from Nude mice to SCID, NOD/SCID, and NOD/SCID rg^null^ mice, and their immunity level has gradually increased. To better simulate human diseases, researchers have constructed the human immune system in immunodeficient mice, and the humanized immune system mouse model provides a powerful tool for studying human diseases. However, there are still many limitations of the various humanized mouse models, and further improvements are needed to truly recapitulate the human immune system. One major hurdle is the scarcity of sources of human cells and tissues, in particular, those obtained from fetal samples carry ethical restrictions. One possible solution to this is induced pluripotent stem cell (iPSC) technology, which enables the use of patient-specific iPSCs allowing a renewable source of autologous cells without immune rejection. The second obstacle is that in humanized mice, secondary lymphoid structures are either missing or disorganized, curtailing essential humoral responses, resulting in impairments for both class switching and affinity maturation post-immunization. To overcome this, lymphoid tissue inducer cells should be introduced without affecting IL2rg receptors. Alternatively, immunodeficient mice can be engrafted with both fetal liver and cells that support fetal liver cell growth from the same clinical donor and supplemented with cytokines, to ensure that the differentiation and maturation of HSCs can take place to improve functional immune cells including macrophages, follicular DC, and T helper cell reconstitution. The third obstacle is that an absence of essential human cytokines hinders optimal HSC engraftment, differentiation, and maturation of functional immune cells. To tackle this issue, mouse models can be hydrodynamically boosted with plasmids encoding cytokines. Despite this improvement, the binding of human cytokines may be hindered by residual mouse cytokines or may induce mouse cells to proliferate and displace the engraftment of human cells due to the cross-reactivity between human and mouse cytokines. Eliminating this problem would require the absolute depletion of murine cells or the introduction of high-affinity human-specific cytokines and growth factors. The fourth hurdle is that human cell engraftment is being negatively affected by mouse cells (red blood cells and innate immune cells) that were not completely depleted during the construction of immunodeficient mice. To improve this, additional gene knock-outs could be added to current strains of immunodeficient mice to further reduce mouse red blood cells, granulocytes, and macrophage functions. However, because of the low human erythrocyte engraftment, excessive reduction of mouse red blood cells might result in anemic mice which have short lifespans, are weak, and are not suitable for experiments. A long-term solution would be to optimize and increase the engraftment rate of human red blood cells in humanized mice so that all traces of mouse red blood cells can be removed. The fifth is the irreproducibility of mouse studies when donors are different for each “batch” of mice. This may be the most important and challenging task for the development of humanized mouse models. Indeed, there is a significant lack of evaluation criteria for donors including clinical data of patients in different disease states and the quantity and quality of their donated specific immune cells. For humanized mouse models, various systemic characteristics are still needed to comment on the development of a successful model. In summary, despite great progress and advances, there are still many limitations to the various humanized mouse models, and further improvements are needed to truly recapitulate the human immune system.

## Author contributions

JC and SL wrote the manuscript. ZX and QRP designed the figures. XW, KS, and SW designed the tables. LY, FG, H-FL, and QJP revised the manuscript. All authors contributed to the article and approved the submitted version.

## Funding

This study was supported by the National Natural Science Foundation of China (no.82070757, 81471530), the Department of established positions for the Zhujiang Scholar from Guangdong Medical University, and Guangdong Basic and Applied Basic Research Foundation (no.2019A1515012203).

## Conflict of interest

The authors declare that the research was conducted in the absence of any commercial or financial relationships that could be construed as a potential conflict of interest.

## Publisher’s note

All claims expressed in this article are solely those of the authors and do not necessarily represent those of their affiliated organizations, or those of the publisher, the editors and the reviewers. Any product that may be evaluated in this article, or claim that may be made by its manufacturer, is not guaranteed or endorsed by the publisher.

## References

[1] Flanagan SP. 'Nude', a new hairless gene with pleiotropic effects in the mouse. Genetical Res (1966) 8:295–309. doi: 10.1017/s0016672300010168 5980117

[2] Blunt T Finnie NJ Taccioli GE Smith GC Demengeot J Gottlieb TM. Defective DNA-dependent protein kinase activity is linked to V(D)J recombination and DNA repair defects associated with the murine scid mutation. Cell (1995) 80:813–23. doi: 10.1016/0092-8674(95)90360-7 7889575

[3] Bosma GC Custer RP Bosma MJ. A severe combined immunodeficiency mutation in the mouse. Nature (1983) 301:527–30. doi: 10.1038/301527a0 6823332

[4] Priestley A Beamish HJ Gell D Amatucci AG Muhlmann-Diaz MC Singleton BK. Molecular and biochemical characterisation of DNA-dependent protein kinase-defective rodent mutant irs-20. Nucleic Acids Res (1998) 26:1965–73. doi: 10.1093/nar/26.8.1965 PMC147487 9518490

[5] Shultz LD Schweitzer PA Christianson SW Gott B Schweitzer IB Tennent B. Multiple defects in innate and adaptive immunologic function in NOD/LtSz-scid mice. J Immunol (Baltimore Md. 1950) (1995) 154:180–91. 7995938

[6] Katano I Ito R Kamisako T Eto T Ogura T Kawai K. NOD-Rag2null IL-2Rγnull mice: an alternative to NOG mice for generation of humanized mice. Exp Anim (2014) 63:321–30. doi: 10.1538/expanim.63.321 PMC4206736 25077762

[7] Pantelouris EM Hair J. Thymus dysgenesis in nude (nu nu) mice. J embryol Exp morphol (1970) 24:615–23. doi: 10.1242/dev.24.3.615 5493276

[8] Tani N Kuchiba K Osada T Watanabe Y Umemoto T. Effect of T-cell deficiency on the formation of periapical lesions in mice: histological comparison between periapical lesion formation in BALB/c and BALB/c nu/nu mice. J endodontics (1995) 21:195–9. doi: 10.1016/s0099-2399(06)80565-0 7673820

[9] Morikawa K Walker SM Nakajima M Pathak S Jessup JM Fidler IJ. Influence of organ environment on the growth, selection, and metastasis of human colon carcinoma cells in nude mice. Cancer Res (1988) 48:6863–71. 2846163

[10] Velasquez LG Galuppo MK Rezende ED Brandão WN Peron JP Uliana SR. Distinct courses of infection with leishmania (L.) amazonensis are observed in BALB/c, BALB/c nude and C57BL/6 mice. Parasitology (2016) 143:692–703. doi: 10.1017/s003118201600024x 26892342

[11] Ganick DJ Sarnwick RD Shahidi NT Manning DD. Inability of intravenously injected monocellular suspensions of human bone marrow to establish in the nude mouse. Int Arch Allergy Appl Immunol (1980) 62:330–3. doi: 10.1159/000232530 6993372

[12] Zhang B Duan Z Zhao Y. Mouse models with human immunity and their application in biomedical research. J Cell Mol Med (2009) 13:1043–58. doi: 10.1111/j.1582-4934.2008.00347.x PMC4496103 18419795

[13] Shultz LD Ishikawa F Greiner DL. Humanized mice in translational biomedical research. Nat Rev Immunol (2007) 7:118–30. doi: 10.1038/nri2017 17259968

[14] Greiner DL Hesselton RA Shultz LD. SCID mouse models of human stem cell engraftment. Stem Cells (Dayton Ohio) (1998) 16:166–77. doi: 10.1002/stem.160166 9617892

[15] Mombaerts P Iacomini J Johnson RS Herrup K Tonegawa S Papaioannou VE. RAG-1-deficient mice have no mature b and T lymphocytes. Cell (1992) 68:869–77. doi: 10.1016/0092-8674(92)90030-g 1547488

[16] Bosma GC Fried M Custer RP Carroll A Gibson DM Bosma MJ. Evidence of functional lymphocytes in some (leaky) scid mice. J Exp Med (1988) 167:1016–33. doi: 10.1084/jem.167.3.1016 PMC2188881 3280724

[17] Shinkai Y Rathbun G Lam KP Oltz EM Stewart V Mendelsohn M. RAG-2-deficient mice lack mature lymphocytes owing to inability to initiate V(D)J rearrangement. Cell (1992) 68:855–67. doi: 10.1016/0092-8674(92)90029-c 1547487

[18] Shultz LD Banuelos S Lyons B Samuels R Burzenski L Gott B. NOD/LtSz-Rag1nullPfpnull mice: a new model system with increased levels of human peripheral leukocyte and hematopoietic stem-cell engraftment. Transplantation (2003) 76:1036–42. doi: 10.1097/01.Tp.0000083041.44829.2c 14557749

[19] Walsh NC Kenney LL Jangalwe S Aryee KE Greiner DL Brehm MA. Humanized mouse models of clinical disease. Annu Rev Pathol (2017) 12:187–215. doi: 10.1146/annurev-pathol-052016-100332 27959627 PMC5280554

[20] Chambers BJ Ljunggren HG. Unique features of NK cell development during ontogeny revealed in studies of RAG-1-deficient mice. Immunol Cell Biol (2010) 88:105–6. doi: 10.1038/icb.2009.103 20010913

[21] Andrews DM Smyth MJ. A potential role for RAG-1 in NK cell development revealed by analysis of NK cells during ontogeny. Immunol Cell Biol (2010) 88:107–16. doi: 10.1038/icb.2009.94 19949422

[22] Makino S Kunimoto K Muraoka Y Mizushima Y Katagiri K Tochino Y. Breeding of a non-obese, diabetic strain of mice. Jikken dobutsu. Exp Anim (1980) 29:1–13. doi: 10.1538/expanim1978.29.1_1 6995140

[23] Hesselton RM Greiner DL Mordes JP Rajan TV Sullivan JL Shultz LD. High levels of human peripheral blood mononuclear cell engraftment and enhanced susceptibility to human immunodeficiency virus type 1 infection in NOD/LtSz-scid/scid mice. J Infect Dis (1995) 172:974–82. doi: 10.1093/infdis/172.4.974 7561218

[24] Wahl A Victor Garcia J. The use of BLT humanized mice to investigate the immune reconstitution of the gastrointestinal tract. J Immunol Methods (2014) 410:28–33. doi: 10.1016/j.jim.2014.06.009 24952245 PMC4163067

[25] Deruaz M Murooka TT Ji S Gavin MA Vrbanac VD Lieberman J. Chemoattractant-mediated leukocyte trafficking enables HIV dissemination from the genital mucosa. JCI Insight (2017) 2:e88533. doi: 10.1172/jci.insight.88533 28405607 PMC5374062

[26] Islas-Ohlmayer M Padgett-Thomas A Domiati-Saad R Melkus MW Cravens PD Martin MP. Experimental infection of NOD/SCID mice reconstituted with human CD34+ cells with Epstein-Barr virus. J Virol (2004) 78(24):13891–900. doi: 10.1128/jvi.78.24.13891-13900.2004 PMC533956 15564497

[27] Miyoshi H Smith KA Mosier DE Verma IM Torbett BE. Transduction of human CD34+ cells that mediate long-term engraftment of NOD/SCID mice by HIV vectors. Sci (New York N.Y.) (1999) 283:682–6. doi: 10.1126/science.283.5402.682 9924027

[28] Takahashi T Katano I Ito R Goto M Abe H Mizuno S. Enhanced antibody responses in a novel NOG transgenic mouse with restored lymph node organogenesis. Front Immunol (2017) 8:2017. doi: 10.3389/fimmu.2017.02017 29387068 PMC5776085

[29] Ito M Hiramatsu H Kobayashi K Suzue K Kawahata M Hioki K. NOD/SCID/gamma(c)(null) mouse: an excellent recipient mouse model for engraftment of human cells. Blood (2002) 100:3175–82. doi: 10.1182/blood-2001-12-0207 12384415

[30] Shultz LD Lyons BL Burzenski LM Gott B Chen X Chaleff S. Human lymphoid and myeloid cell development in NOD/LtSz-scid IL2R gamma null mice engrafted with mobilized human hemopoietic stem cells. J Immunol (Baltimore Md. 1950) (2005) 174:6477–89. doi: 10.4049/jimmunol.174.10.6477 15879151

[31] Pearson T Shultz LD Miller D King M Laning J Fodor W. Non-obese diabetic-recombination activating gene-1 (NOD-Rag1 null) interleukin (IL)-2 receptor common gamma chain (IL2r gamma null) null mice: a radioresistant model for human lymphohaematopoietic engraftment. Clin Exp Immunol (2008) 154:270–84. doi: 10.1111/j.1365-2249.2008.03753.x PMC2612717 18785974

[32] Brooks DG Kitchen SG Kitchen CM Scripture-Adams DD Zack JA. Generation of HIV latency during thymopoiesis. Nat Med (2001) 7:459–64. doi: 10.1038/86531 11283673

[33] Brehm MA Cuthbert A Yang C Miller DM DiIorio P Laning J. Parameters for establishing humanized mouse models to study human immunity: analysis of human hematopoietic stem cell engraftment in three immunodeficient strains of mice bearing the IL2rgamma(null) mutation. Clin Immunol (Orlando Fla.) (2010) 135:84–98. doi: 10.1016/j.clim.2009.12.008 PMC2835837 20096637

[34] Machida K Suemizu H Kawai K Ishikawa T Sawada R Ohnishi Y. Higher susceptibility of NOG mice to xenotransplanted tumors. J toxicol Sci (2009) 34:123–7. doi: 10.2131/jts.34.123 19182442

[35] King MA Covassin L Brehm MA Racki W Pearson T Leif J. Human peripheral blood leucocyte non-obese diabetic-severe combined immunodeficiency interleukin-2 receptor gamma chain gene mouse model of xenogeneic graft-versus-host-like disease and the role of host major histocompatibility complex. Clin Exp Immunol (2009) 157:104–18. doi: 10.1111/j.1365-2249.2009.03933.x PMC2710598 19659776

[36] Wunderlich M Chou FS Link KA Mizukawa B Perry RL Carroll M. AML xenograft efficiency is significantly improved in NOD/SCID-IL2RG mice constitutively expressing human SCF, GM-CSF and IL-3. Leukemia (2010) 24:1785–8. doi: 10.1038/leu.2010.158 PMC5439963 20686503

[37] Zhang L Meissner E Chen J Su L. Current humanized mouse models for studying human immunology and HIV-1 immuno-pathogenesis. Sci China. Life Sci (2010) 53:195–203. doi: 10.1007/s11427-010-0059-7 20596827 PMC4224686

[38] Tanaka S Saito Y Kunisawa J Kurashima Y Wake T Suzuki N. Development of mature and functional human myeloid subsets in hematopoietic stem cell-engrafted NOD/SCID/IL2rγKO mice. J Immunol (Baltimore Md. 1950) (2012) 188:6145–55. doi: 10.4049/jimmunol.1103660 PMC3370073 22611244

[39] Oshima K Ruhul Amin AR Suzuki A Hamaguchi M Matsuda S. SHPS-1, a multifunctional transmembrane glycoprotein. FEBS Lett (2002) 519:1–7. doi: 10.1016/s0014-5793(02)02703-5 12023008

[40] Barclay AN Brown MH. The SIRP family of receptors and immune regulation. Nat Rev Immunol (2006) 6:457–64. doi: 10.1038/nri1859 16691243

[41] Matozaki T Murata Y Okazawa H Ohnishi H. Functions and molecular mechanisms of the CD47-SIRPalpha signalling pathway. Trends Cell Biol (2009) 19:72–80. doi: 10.1016/j.tcb.2008.12.001 19144521

[42] Tsai RK Discher DE. Inhibition of "self" engulfment through deactivation of myosin-II at the phagocytic synapse between human cells. J Cell Biol (2008) 180:989–1003. doi: 10.1083/jcb.200708043 18332220 PMC2265407

[43] Legrand N Huntington ND Nagasawa M Bakker AQ Schotte R Strick-Marchand H. Functional CD47/signal regulatory protein alpha (SIRP(alpha)) interaction is required for optimal human T- and natural killer- (NK) cell homeostasis *in vivo* . Proc Natl Acad Sci United States America (2011) 108:13224–9. doi: 10.1073/pnas.1101398108 PMC3156191 21788504

[44] Takenaka K Prasolava TK Wang JC Mortin-Toth SM Khalouei S Gan OI. Polymorphism in sirpa modulates engraftment of human hematopoietic stem cells. Nat Immunol (2007) 8(12):1313–23. doi: 10.1038/ni1527 17982459

[45] Yamauchi T Takenaka K Urata S Shima T Kikushige Y Tokuyama T. Polymorphic sirpa is the genetic determinant for NOD-based mouse lines to achieve efficient human cell engraftment. Blood (2013) 121:1316–25. doi: 10.1182/blood-2012-06-440354 23293079

[46] Li Y Masse-Ranson G Garcia Z Bruel T Kök A Strick-Marchand H. A human immune system mouse model with robust lymph node development. Nat Methods (2018) 15:623–30. doi: 10.1038/s41592-018-0071-6 30065364

[47] Andrade D Redecha PB Vukelic M Qing X Perino G Salmon JE. Engraftment of peripheral blood mononuclear cells from systemic lupus erythematosus and antiphospholipid syndrome patient donors into BALB-RAG-2-/- IL-2Rγ-/- mice: a promising model for studying human disease. Arthritis rheumatism (2011) 63:2764–73. doi: 10.1002/art.30424 PMC3168580 21560114

[48] Chen J Liao S Zhou H Yang L Guo F Chen S. Humanized mouse models of systemic lupus erythematosus: Opportunities and challenges. Front Immunol (2021) 12:816956. doi: 10.3389/fimmu.2021.816956 35116040 PMC8804209

[49] Akkina R. Humanized mice for studying human immune responses and generating human monoclonal antibodies. Microbiol Spectr (2014) 2(2):0003–2012. doi: 10.1128/microbiolspec.AID-0003-2012 26105817

[50] Watanabe Y Takahashi T Okajima A Shiokawa M Ishii N Katano I. The analysis of the functions of human b and T cells in humanized NOD/shi-scid/gammac(null) (NOG) mice (hu-HSC NOG mice). Int Immunol (2009) 21:843–58. doi: 10.1093/intimm/dxp050 19515798

[51] Takahama Y Nitta T Mat Ripen A Nitta S Murata S Tanaka K. Role of thymic cortex-specific self-peptides in positive selection of T cells. Semin Immunol (2010) 22:287–93. doi: 10.1016/j.smim.2010.04.012 20510627

[52] Fairfax KA Kallies A Nutt SL Tarlinton DM. Plasma cell development: from b-cell subsets to long-term survival niches. Semin Immunol (2008) 20:49–58. doi: 10.1016/j.smim.2007.12.002 18222702

[53] Danner R Chaudhari SN Rosenberger J Surls J Richie TL Brumeanu TD. Expression of HLA class II molecules in humanized NOD.Rag1KO.IL2RgcKO mice is critical for development and function of human T and b cells. PloS One (2011) 6:e19826. doi: 10.1371/journal.pone.0019826 21611197 PMC3096643

[54] Ito K Bian HJ Molina M Han J Magram J Saar E. HLA-DR4-IE chimeric class II transgenic, murine class II-deficient mice are susceptible to experimental allergic encephalomyelitis. J Exp Med (1996) 183:2635–44. doi: 10.1084/jem.183.6.2635 PMC2192625 8676084

[55] Yue X Petersen F Shu Y Kasper B Magatsin JDT Ahmadi M. Transfer of PBMC from SSc patients induces autoantibodies and systemic inflammation in Rag2-/-/IL2rg-/- mice. Front Immunol (2021) 12:677970. doi: 10.3389/fimmu.2021.677970 34248959 PMC8261241

[56] Ehx G Somja J Warnatz HJ Ritacco C Hannon M Delens L. Xenogeneic graft-Versus-Host disease in humanized NSG and NSG-HLA-A2/HHD mice. Front Immunol (2018) 9:1943. doi: 10.3389/fimmu.2018.01943 30214443 PMC6125392

[57] Lin S Huang G Cheng L Li Z Xiao Y Deng Q. Establishment of peripheral blood mononuclear cell-derived humanized lung cancer mouse models for studying efficacy of PD-L1/PD-1 targeted immunotherapy. mAbs (2018) 10:1301–11. doi: 10.1080/19420862.2018.1518948 PMC6284590 30204048

[58] Koo GC Hasan A O'Reilly RJ. Use of humanized severe combined immunodeficient mice for human vaccine development. Expert Rev Vaccines (2009) 8:113–20. doi: 10.1586/14760584.8.1.113 PMC2677709 19093778

[59] Racki WJ Covassin L Brehm M Pino S Ignotz R Dunn R. NOD-scid IL2rgamma(null) mouse model of human skin transplantation and allograft rejection. Transplantation (2010) 89:527–36. doi: 10.1097/TP.0b013e3181c90242 PMC2901915 20134397

[60] Kumar P Ban HS Kim SS Wu H Pearson T Greiner DL. T Cell-specific siRNA delivery suppresses HIV-1 infection in humanized mice. Cell (2008) 134:577–86. doi: 10.1016/j.cell.2008.06.034 PMC2943428 18691745

[61] Kremsdorf D Strick-Marchand H. Modeling hepatitis virus infections and treatment strategies in humanized mice. Curr Opin Virol (2017) 25:119–25. doi: 10.1016/j.coviro.2017.07.029 28858692

[62] Cui X Snapper CM. Epstein Barr Virus: Development of vaccines and immune cell therapy for EBV-associated diseases. Front Immunol (2021) 12:734471. doi: 10.3389/fimmu.2021.734471 34691042 PMC8532523

[63] Jaggi U Wang S Tormanen K Matundan H Ljubimov AV Ghiasi H. Role of herpes simplex virus type 1 (HSV-1) glycoprotein K (gK) pathogenic CD8(+) T cells in exacerbation of eye disease. Front Immunol (2018) 9:2895. doi: 10.3389/fimmu.2018.02895 30581441 PMC6292954

[64] Crawford LB Streblow DN Hakki M Nelson JA Caposio P. Humanized mouse models of human cytomegalovirus infection. Curr Opin Virol (2015) 13:86–92. doi: 10.1016/j.coviro.2015.06.006 26118890 PMC4599783

[65] Fujiwara S. Animal models of human gammaherpesvirus infections. Adv Exp Med Biol (2018) 1045:413–36. doi: 10.1007/978-981-10-7230-7_19 29896678

[66] Nervi B Rettig MP Ritchey JK Wang HL Bauer G Walker J. Factors affecting human T cell engraftment, trafficking, and associated xenogeneic graft-vs-host disease in NOD/SCID beta2mnull mice. Exp Hematol (2007) 35:1823–38. doi: 10.1016/j.exphem.2007.06.007 PMC2238776 17764813

[67] De La Rochere P Guil-Luna S Decaudin D Azar G Sidhu SS Piaggio E. Humanized mice for the study of immuno-oncology. Trends Immunol (2018) 39:748–63. doi: 10.1016/j.it.2018.07.001 30077656

[68] Brehm MA Kenney LL Wiles MV Low BE Tisch RM Burzenski L. Lack of acute xenogeneic graft- versus-host disease, but retention of T-cell function following engraftment of human peripheral blood mononuclear cells in NSG mice deficient in MHC class I and II expression. FASEB J (2019) 33:3137–51. doi: 10.1096/fj.201800636R PMC6404556 30383447

[69] Billerbeck E Mommersteeg MC Shlomai A Xiao JW Andrus L Bhatta A. Humanized mice efficiently engrafted with fetal hepatoblasts and syngeneic immune cells develop human monocytes and NK cells. J Hepatol (2016) 65:334–43. doi: 10.1016/j.jhep.2016.04.022 PMC4955758 27151182

[70] Cao B Zhang Z Grassinger J Williams B Heazlewood CK Churches QI. Therapeutic targeting and rapid mobilization of endosteal HSC using a small molecule integrin antagonist. Nat Commun (2016) 7:11007. doi: 10.1038/ncomms11007 26975966 PMC4796355

[71] Abarrategi A Foster K Hamilton A Mian SA Passaro D Gribben J. Versatile humanized niche model enables study of normal and malignant human hematopoiesis. J Clin Invest (2017) 127:543–8. doi: 10.1172/jci89364 PMC5272182 28067666

[72] Rongvaux A Takizawa H Strowig T Willinger T Eynon EE Flavell RA. Human hemato-lymphoid system mice: current use and future potential for medicine. Annu Rev Immunol (2013) 31:635–74. doi: 10.1146/annurev-immunol-032712-095921 PMC4120191 23330956

[73] Kim SS Kumar P Ye C Shankar P. Humanized mice for studying human leukocyte integrins *in vivo* . Methods Mol Biol (Clifton N.J.) (2012) 757:509–21. doi: 10.1007/978-1-61779-166-6_30 PMC4310724 21909931

[74] Su H Cheng Y Sravanam S Mathews S Gorantla S Poluektova LY. Immune activations and viral tissue compartmentalization during progressive HIV-1 infection of humanized mice. Front Immunol (2019) 10:340. doi: 10.3389/fimmu.2019.00340 30873181 PMC6403174

[75] Worni-Schudel IM Clark AG Chien T Hwang KK Chen BJ Foster MH. Recovery of a human natural antibody against the noncollagenous-1 domain of type IV collagen using humanized models. J Trans Med (2015) 13:185. doi: 10.1186/s12967-015-0539-4 PMC4467618 26048777

[76] Lepus CM Gibson TF Gerber SA Kawikova I Szczepanik M Hossain J. Comparison of human fetal liver, umbilical cord blood, and adult blood hematopoietic stem cell engraftment in NOD-scid/gammac-/-, balb/c-Rag1-/-gammac-/- and C.B-17-scid/bg immunodeficient mice. Hum Immunol (2009) 70:790–802. doi: 10.1016/j.humimm.2009.06.005 19524633 PMC2949440

[77] Berges BK Rowan MR. The utility of the new generation of humanized mice to study HIV-1 infection: transmission, prevention, pathogenesis, and treatment. Retrovirology (2011) 8:65. doi: 10.1186/1742-4690-8-65 21835012 PMC3170263

[78] Shultz LD Brehm MA Bavari S Greiner DL. Humanized mice as a preclinical tool for infectious disease and biomedical research. Ann New York Acad Sci (2011) 1245:50–4. doi: 10.1111/j.1749-6632.2011.06310.x PMC3514446 22211979

[79] McCune JM. Development and applications of the SCID-hu mouse model. Semin Immunol (1996) 8:187–96. doi: 10.1006/smim.1996.0024 8883141

[80] McCune JM Namikawa R Kaneshima H Shultz LD Lieberman M Weissman IL. The SCID-hu mouse: murine model for the analysis of human hematolymphoid differentiation and function. Sci (New York N.Y.) (1988) 241:1632–9. doi: 10.1126/science.241.4873.1632 2971269

[81] Jamieson BD Zack JA. Murine models for HIV disease. AIDS (London England) (1999) 13:S5–11. 10885758

[82] Lan P Tonomura N Shimizu A Wang S Yang YG. Reconstitution of a functional human immune system in immunodeficient mice through combined human fetal thymus/liver and CD34+ cell transplantation. Blood (2006) 108:487–92. doi: 10.1182/blood-2005-11-4388 16410443

[83] Wege AK Melkus MW Denton PW Estes JD Garcia JV. Functional and phenotypic characterization of the humanized BLT mouse model. Curr topics Microbiol Immunol (2008) 324:149–65. doi: 10.1007/978-3-540-75647-7_10 18481459

[84] Stoddart CA Maidji E Galkina SA Kosikova G Rivera JM Moreno ME. Superior human leukocyte reconstitution and susceptibility to vaginal HIV transmission in humanized NOD-scid IL-2Rγ(-/-) (NSG) BLT mice. Virology (2011) 417:154–60. doi: 10.1016/j.virol.2011.05.013 PMC3152643 21684569

[85] Brainard DM Seung E Frahm N Cariappa A Bailey CC Hart WK. Induction of robust cellular and humoral virus-specific adaptive immune responses in human immunodeficiency virus-infected humanized BLT mice. J Virol (2009) 83:7305–21. doi: 10.1128/jvi.02207-08 PMC2704767 19420076

[86] Lavender KJ Pace C Sutter K Messer RJ Pouncey DL Cummins NW. An advanced BLT-humanized mouse model for extended HIV-1 cure studies. AIDS (London England) (2018) 32:1–10. doi: 10.1097/qad.0000000000001674 PMC5718929 29112072

[87] Bonte S Snauwaert S Vanhee S Dolens AC Taghon T Vandekerckhove B. Humanized mice to study human T cell development. Methods Mol Biol (Clifton N.J.) (2016) 1323:253–72. doi: 10.1007/978-1-4939-2809-5_21 26294414

[88] Bournazos S DiLillo DJ Ravetch JV. Humanized mice to study FcγR function. Curr topics Microbiol Immunol (2014) 382:237–48. doi: 10.1007/978-3-319-07911-0_11 25116103

[89] Brehm MA Jouvet N Greiner DL Shultz LD. Humanized mice for the study of infectious diseases. Curr Opin Immunol (2013) 25:428–35. doi: 10.1016/j.coi.2013.05.012 PMC3775881 23751490

[90] Usmani SM Murooka TT Deruaz M Koh WH Sharaf RR Di Pilato M. HIV-1 balances the fitness costs and benefits of disrupting the host cell actin cytoskeleton early after mucosal transmission. Cell Host Microbe (2019) 25:73–86.e75. doi: 10.1016/j.chom.2018.12.008 30629922 PMC6456338

[91] Smith DJ Lin LJ Moon H Pham AT Wang X Liu S. Propagating humanized BLT mice for the study of human immunology and immunotherapy. Stem Cells Dev (2016) 25:1863–73. doi: 10.1089/scd.2016.0193 PMC5165681 27608727

[92] Vatakis DN Bristol GC Kim SG Levin B Liu W Radu CG. Using the BLT humanized mouse as a stem cell based gene therapy tumor model. J visualized experiments (2012) 18(70):e4181. doi: 10.3791/4181 PMC3576416 23271478

[93] Tournoy KG Depraetere S Pauwels RA Leroux-Roels GG. Mouse strain and conditioning regimen determine survival and function of human leucocytes in immunodeficient mice. Clin Exp Immunol (2000) 119:231–9. doi: 10.1046/j.1365-2249.2000.01099.x PMC1905527 10606988

[94] Santini SM Rizza P Logozzi MA Sestili P Gherardi G Lande R. The SCID mouse reaction to human peripheral blood mononuclear leukocyte engraftment. neutrophil recruitment induced expression of a wide spectrum of murine cytokines and mouse leukopoiesis, including thymic differentiation. Transplantation (1995) 60:1306–14. doi: 10.1097/00007890-199512000-00020 8525526

[95] André MC Erbacher A Gille C Schmauke V Goecke B Hohberger A. Long-term human CD34+ stem cell-engrafted nonobese diabetic/SCID/IL-2R gamma(null) mice show impaired CD8+ T cell maintenance and a functional arrest of immature NK cells. J Immunol (Baltimore Md. 1950) (2010) 185:2710–20. doi: 10.4049/jimmunol.1000583 20668220

[96] Cashman JD Eaves CJ. Human growth factor-enhanced regeneration of transplantable human hematopoietic stem cells in nonobese diabetic/severe combined immunodeficient mice. Blood (1999) 93:481–7. doi: 10.1182/blood.V93.2.481 9885209

[97] Ding Y Wilkinson A Idris A Fancke B O'Keeffe M Khalil D. FLT3-ligand treatment of humanized mice results in the generation of large numbers of CD141+ and CD1c+ dendritic cells *in vivo* . J Immunol (Baltimore Md. 1950) (2014) 192:1982–9. doi: 10.4049/jimmunol.1302391 24453245

[98] Pek EA Chan T Reid S Ashkar AA. Characterization and IL-15 dependence of NK cells in humanized mice. Immunobiology (2011) 216:218–24. doi: 10.1016/j.imbio.2010.04.008 20627447

[99] O'Connell RM Balazs AB Rao DS Kivork C Yang L Baltimore D. Lentiviral vector delivery of human interleukin-7 (hIL-7) to human immune system (HIS) mice expands T lymphocyte populations. PloS One (2010) 5:e12009. doi: 10.1371/journal.pone.0012009 20700454 PMC2917362

[100] Chen Q Khoury M Chen J. Expression of human cytokines dramatically improves reconstitution of specific human-blood lineage cells in humanized mice. Proc Natl Acad Sci United States America (2009) 106:21783–8. doi: 10.1073/pnas.0912274106 PMC2789167 19966223

[101] Rongvaux A Willinger T Martinek J Strowig T Gearty SV Teichmann LL. Development and function of human innate immune cells in a humanized mouse model. Nat Biotechnol (2014) 32:364–72. doi: 10.1038/nbt.2858 PMC4017589 24633240

[102] Saito Y Ellegast JM Rafiei A Song Y Kull D Heikenwalder M. Peripheral blood CD34(+) cells efficiently engraft human cytokine knock-in mice. Blood (2016) 128:1829–33. doi: 10.1182/blood-2015-10-676452 PMC5054696 27543436

[103] Ito R Takahashi T Katano I Kawai K Kamisako T Ogura T. Establishment of a human allergy model using human IL-3/GM-CSF-transgenic NOG mice. J Immunol (Baltimore Md. 1950) (2013) 191:2890–9. doi: 10.4049/jimmunol.1203543 23956433

[104] Rongvaux A Willinger T Takizawa H Rathinam C Auerbach W Murphy AJ. Human thrombopoietin knock-in mice efficiently support human hematopoiesis *in vivo* . Proc Natl Acad Sci United States America (2011) 108:2378–83. doi: 10.1073/pnas.1019524108 PMC3038726 21262827

[105] Billerbeck E Barry WT Mu K Dorner M Rice CM Ploss A. Development of human CD4+FoxP3+ regulatory T cells in human stem cell factor-, granulocyte-macrophage colony-stimulating factor-, and interleukin-3-expressing NOD-SCID IL2Rγ(null) humanized mice. Blood (2011) 117:3076–86. doi: 10.1182/blood-2010-08-301507 PMC3062310 21252091

[106] Miller PH Cheung AM Beer PA Knapp DJ Dhillon K Rabu G. Enhanced normal short-term human myelopoiesis in mice engineered to express human-specific myeloid growth factors. Blood (2013) 121:e1–4. doi: 10.1182/blood-2012-09-456566 23233660

[107] Sherr CJ Rettenmier CW Roussel MF. Macrophage colony-stimulating factor, CSF-1, and its proto-oncogene-encoded receptor. Cold Spring Harbor Symp quantitative Biol (1988) 53:521–30. doi: 10.1101/sqb.1988.053.01.060 2855492

[108] Sefik E Qu R Junqueira C Kaffe E Mirza H Zhao J. Inflammasome activation in infected macrophages drives COVID-19 pathology. Nature (2022) 606:585–93. doi: 10.1038/s41586-022-04802-1 PMC9288243 35483404

[109] Giuliani AL Wiener E Lee MJ Brown IN Berti G Wickramasinghe SN. Changes in murine bone marrow macrophages and erythroid burst-forming cells following the intravenous injection of liposome-encapsulated dichloromethylene diphosphonate (Cl2MDP). Eur J haematol (2001) 66:221–9. doi: 10.1034/j.1600-0609.2001.066004221.x 11380601

[110] Barclay AN Van den Berg TK. The interaction between signal regulatory protein alpha (SIRPα) and CD47: structure, function, and therapeutic target. Annu Rev Immunol (2014) 32:25–50. doi: 10.1146/annurev-immunol-032713-120142 24215318

[111] Vandenberg O Martiny D Rochas O van Belkum A Kozlakidis Z. Considerations for diagnostic COVID-19 tests. Nat Rev Microbiol (2021) 19:171–83. doi: 10.1038/s41579-020-00461-z PMC7556561 33057203

[112] Chen Y Klein SL Garibaldi BT Li H Wu C Osevala NM. Aging in COVID-19: Vulnerability, immunity and intervention. Ageing Res Rev (2021) 65:101205. doi: 10.1016/j.arr.2020.101205 33137510 PMC7604159

[113] Taleghani N Taghipour F. Diagnosis of COVID-19 for controlling the pandemic: A review of the state-of-the-art. Biosensors bioelectronics (2021) 174:112830. doi: 10.1016/j.bios.2020.112830 33339696 PMC7694563

